# Recent Developments in HLA B27 Anterior Uveitis

**DOI:** 10.3389/fimmu.2020.608134

**Published:** 2021-01-05

**Authors:** Denis Wakefield, Daniel Clarke, Peter McCluskey

**Affiliations:** ^1^ Faculty of Medicine, University of NSW Sydney, Kensington, NSW, Australia; ^2^ NSW Health Pathology and South Eastern Sydney, LHD, Sydney, NSW, Australia; ^3^ Department of Medicine, South Eastern Sydney, LHD, Sydney, NSW, Australia; ^4^ Save Sight Institute, The University of Sydney, Sydney, NSW, Australia; ^5^ Discipline of Clinical Ophthalmology and Eye Health, Faculty of Medicine and Health, The University of Sydney, Sydney, NSW, Australia

**Keywords:** acute anterior uveitis, HLA B27, pathogenesis, microbiome, review

## Abstract

There has been steady progress in understanding the pathogenesis, clinical features, and effective treatment of acute anterior uveitis (AU) over the past 5 years. Large gene wide association studies have confirmed that AU is a polygenic disease, with overlaps with the seronegative arthropathies and inflammatory bowel diseases, associations that have been repeatedly confirmed in clinical studies. The role of the microbiome in AU has received increased research attention, with recent evidence indicating that human leukocyte antigen B27 (HLA B27) may influence the composition of the gut microbiome in experimental animals. Extensive clinical investigations have confirmed the typical features of acute AU (AAU) and its response to topical, regional and systemic immunosuppressive treatment. Increased understanding of the role of cytokines has resulted in studies confirming the value of anti-cytokine therapy [anti–tumor necrosis factor (anti-TNF) and interleukin 6 (IL-6) therapy] in severe and recurrent cases of AAU, particularly in subjects with an associated spondyloarthopathy (SpA) and in juvenile idiopathic arthritis (JIA)–associated AAU.

## Introduction

Uveitis is a common vision threatening inflammatory eye disease with a reported annual incidence of between 17 and 52 per 100,000 and prevalence of between 38 and 370 per 100,000 of the population (1–4). The disease is most common in children, young and middle-aged adults. Uveitis and its complications account for up to 10% of those with severe vision impairment and blindness (5). Anterior uveitis (AU) is present in up to 90% of cases of uveitis and approximately 50% of all patients with acute AU (AAU) are human leukocyte antigen B27 (HLA B27) positive. HLA B27 is also strongly associated with ankylosing spondylitis (AS), reactive arthritis (ReA), and other spondyloarthropathies (SpA) (6). These associations are among the strongest of any HLA antigen to a human disease, although the pathogenetic mechanism(s) involved remain unknown (7). There has been relatively little research devoted to the immunopathogenesis of HLA B27 AAU, compared with the numerous studies of the immunopathogenesis of the SpA’s.

Evidence that HLA B27 is directly involved in the pathogenesis of this group of diseases is supported by the fact that HLA B27 is associated with AAU and SpA in many populations and ethnic groups, in the context of over 140 haplotypes (the HLA B2705 subtype is the most common in Caucasians). Secondly, some HLA B27 subtypes are not associated to AAU or SpA, suggesting that subtype polymorphism modulate disease susceptibility, and thirdly, HLA B27 transgenic rats and mice develop a disease that strongly resembles human SpA, although these animals do not spontaneously develop AAU (8).

## Clinical Features of HLA B27 Anterior Uveitis

In a large study of over 2000 patients with axial SpA from 83 rheumatology centers in the United Kingdom 23.5% of the patients in the cohort reported at least 1 episode of AAU. The mean age of patients was 51 years with a male to female ratio of 2:1. The factors associated with increased prevalence of AAU in this cohort were HLA B27 positivity, university education, age, and the presence and duration of axial spondylo-arthritis (9). There is a well-defined clinical phenotype for patients with AAU associated with spondyloarthritis which was confirmed in a study by Peizeng et al. in a large Chinese population (10). Typical symptoms include: acute onset of blurred vision, ocular redness, pain and photophobia. Important clinical descriptors include: unilateral episodes, alternation between eyes, sudden onset and limited duration. HLA B27 AAU is non-granulomatous and characterized by flare and cells in the anterior chamber that commonly spills over into the anterior vitreous. Other clinical features such as hypopyon and posterior synechiae may occur. HLA B27 associated uveitis is nearly always confined to the anterior uvea as AAU, although uncommonly there can be prominent intermediate uveitis and involvement of the posterior segment (11). A Chinese study also found that asymptomatic previously unrecognized, retinal vascular involvement often involving the peripheral retina is not uncommon (10). HLA B27 associated AAU is further characterized by recurrences, which are more frequent early in the course of the disease and become less frequent with the duration of disease (12).

Severe and/or recurrent episodes of AAU may result in structural complications such as cataract, elevated intraocular pressure, glaucoma and cystoid macular oedema, which become increasingly common with multiple episodes of uveitis (13). Some patients develop chronic AU following very severe attacks or frequent recurrences. D’Ambrosia and colleagues performed a literature-based meta-analysis to characterize the clinical features and complications associated with HLA B27 AU. The results confirmed the strong association of AU with AS, increased male prevalence, unilateral alternating episodes of uveitis often characterized by a fibrinous inflammatory reaction with or without hypopyon, and occasionally optic disc swelling. The prevalence of raised intraocular pressure and glaucoma were more common in the HLA B27 negative cases of AAU. Interestingly, they concluded that there is no significant difference between HLA B27 positive and HLA B27 negative AAU with regard to final visual acuity and structural complications, such as posterior synechiae, cataract, and macular oedema (14).

## Genetics and Anterior Uveitis

The heritability of HLA B27 AAU has a strong association with the major histocombatibility complex (MHC) type 1 allele which over 50% of those with primary AAU possess (7). In an examination of 152 first degree relatives of 42 randomly selected HLA B27 AAU patients, 6% were found to develop the condition (15). The same Dutch study also identified that there may be other genetic factors influencing inheritance, with higher rates of proband first degree relatives developing AAU than in the normal HLA B27 population (15). Recent genotyping studies have supported this observation with several non MHC loci demonstrating genome wide association significance. In one study comparing AAU subjects to healthy controls using the Illumina Immunochip, associations were established with ERAP1, IL23R, and the intergenic region 2p15 (16).

The first genome wide association study (GWAS) of patients with AAU with or without AS identified new susceptibility genes and confirmed the strong association of HLA B27 AAU with SpA (17). Gene associations were observed for 11 loci, including the previously reported ERAP1 and NOS2. Associations were also found at previously unrecognized sites for AS and AU including MERTK, KIFAP3, CLCN 7, ACAA2, and five intergenic loci (17). A number of genetic factors critical to the immune response to microorganisms, such as IL10, IL18R1-IL1R1, IL6R, and KIF21B, were previously reported in a GWAS study of patients with AS and AAU (16).

Despite the association of the ERAP1 gene with HLA B27 SpA and AAU the role of this gene in peptide selection and processing in the pathogenesis of these diseases has not been defined. Barnea et al. examined the effect of ERAP1 deletion in HLA B27 transgenic rats. The male HLA B27 transgenic rats develop a spondylo-arthritic type of disease, but not AU, and have been used as a model of spondylo-arthritis. Deletion of the ERAP1 gene did not affect the development of SpA (18). Interestingly, examination of the peptidomes of rats susceptible to arthritis was similar to those of non-susceptible rats and no peptides were found to be uniquely associated with arthritis.

Using next generation sequencing to identify T-cell receptor beta motifs and amino acid sequences specific to HLA B27 positive patients with AS. Faham et al. recently reported sequences specific to AS, indicating that specific antigens possibly related to the pathogenesis of AS may be identifiable using such an approach (19). Such studies have not been reported in patients with HLA B27 associated AAU but could yield results strengthening the argument that CD8 positive T cells are pivotal in the pathogenesis of this disease.

## Infection and HLA B27 AAU

There is considerable epidemiological, clinical, and experimental evidence in patients with AU implicating the role of microbial infections as part of the molecular mimicry or arthritogenic peptide hypothesis. In particular, infection with *Chlamydia Trachomatis* and gram negative bacteria including *Klebsiella*, *Salmonella*, *Yersinia*, *Shigella* species, and *Campylobacter jejuni*, has been reported to trigger AU and SpA (20–22). Furthermore, there is compelling evidence implicating *Chlamydia trachomatis* in the pathogenesis of ReA and undifferentiated spondylarthroses (19, 20, 22–24). More recently, epidemiological evidence has emerged implicating *Ureaplasma Urealyticum* (25) and a yet to be identified fungus (26) with the occurrence of uveitis. We have previously provided evidence for the role of microbial triggers in the pathogenesis of HLA B27 AAU, by demonstrating humoral and cellular immune responses to *C. trachomatis* and *Yersinia* (26, 27).

Schofield et al. have delineated potential inciting peptide sequences common between enteric bacteria capsules and the third hypervariable region of the HLA B27 molecule (28). Further studies in this vein have found that *Klebsiella* nitrogenase shares six consecutive amino-acids with HLA B27 (29) and surface proteins of *Salmonella*, *Yersinia*, and *Shigella* have homologous sequences (30). Investigating mice models of disease Horai and colleagues demonstrated that the presence of enteric bacteria triggers retinal specific auto reactive T cells and subsequently spontaneous uveitis (31). Unfortunately, attempts to further localize the effect to self-peptides or self-antigens have thus far failed.

Schittenhelm and colleagues have searched for the existence of disease inducing peptides, the so-called arthrogenic or uveitic peptide(s) proposed to be important in the pathogenesis of HLA B27 related diseases (32). Using sophisticated Mass Spectroscopy they examined 1,500 epitopes across several different HLA class I allotypes (HLA B27:02- HLA B27:09). Analysis of this large data set identified 26 peptides, which were present in lower abundance by non-disease-related HLA B27 allotypes compared with the disease associated HLA B27 subtypes. It is yet to be ascertained whether these peptides represent the arthrogenic peptide proposed to cause SpA’s. The high amount of overlap found in the absolute peptide binding preferences of the HLA B27 allotypes (:02 to :09) has shifted the focus of the arthritogenic peptide hypothesis to quantitative analysis of peptide binding conformation, self-peptide presentation and T cell activation (33). It would be of great interest to use similar technology to identify potential peptides related to HLA B27 AU.

## The Role of the Microbiome

There is considerable recent interest in the role of the microbiome in influencing immune responses and susceptibility to diseases such as Crohn’s disease, ulcerative colitis, SpA’s, and uveitis (34–36). Studies in HLA B27 transgenic rats demonstrate an altered microbiome compared to non-transgenic animals (37). The mechanisms by which the microbiome may influence immune responses are poorly understood. One theory is that gut microbes or their products may alter the permeability of the gut wall allowing translocation of bacteria and/or microbial products to regional lymph nodes, which generate a systemic immune response. Such increased gut permeability has been described in patients with AS and inflammatory bowel disease. Patient with ReA have had microbial antigens detected in joint fluid and subsequently been shown to have a clinical response to antibiotic therapy (38). Additionally, endotoxin, which is a component of Gram negative bacteria cell-walls has been shown in experimental animals to induce AU when injected into the foot pad of rodents (39). Similarly, we have shown that patients with active AU have increased expression of Toll like receptors 4 (which binds endotoxin) in the peripheral blood during acute attacks of AU (40). An alternative theory holds that there is molecular mimicry between microbial and self-antigens. Such micro-organisms and their associated antigens may generate an immune response whereby antibodies or T cells cross-reacts with self-antigens as is known to occur in diseases such as rheumatic fever and post-infection Gilliam Barré syndrome (41). Thirdly, it has been shown that the microbiome may affect the immune response by influencing T cells to synthesize increased amounts of IL-17 and the number of regulatory T cells that express the transcription factor fox P3 (42). Furthermore, it has been shown that a large number of microbial peptides bind the HLA B27 molecule and such microbial peptides may induce an immune response in certain target organs such as the eye and joint (43). We have recently shown, for example, that peptides derived from Chlamydia trachomatis bind the HLA B27 molecule and when such peptides are injected with adjuvant into experimental animals they induce an arthritis and less frequently AU (27).

An immune response to peptides derived from gut microorganisms (microbiome) could cross-react with endogenous peptides leading to the development of AU (34). This concept is strengthened by the results of the recent studies indicating that a large number of peptides derived from microorganisms bind to the HLA B27 molecule as well as our studies showing increased affinity of chlamydia and derived peptides for the HLA B27 molecule (27, 28, 44–46). We have also shown that immunizing rats with chlamydia derived peptides leads to a polyarthritis and occasional uveitis in these animals (47). Furthermore, a peptide (C34) derived from type VI collagen was shown to be recognized by CD8^+^ T cells from patients with AS (48). Additionally, cross-reactivity between *chlamydial* peptides and homologous peptides from the heart muscle-specific protein -myosin was shown to be involved in the pathogenesis of autoimmune myocarditis in mice (49).

The microbiome of the aqueous humor has not been examined in patients with acute and recurrent AU. The anterior chamber of the eye is usually considered to be sterile, although a recent study indicated that in fact the aqueous humor may have a microbiome that was previously unrecognized (50). Examination of the aqueous humor for evidence of microorganisms during acute attacks of AU could reveal valuable information with regard to the microbial and immunological mechanisms underlying this common inflammatory disease. If the gut microbiome does play a key role in diseases induction, a variety of therapeutic strategies could be developed to alter the microbiome or the immune response to particular microorganisms implicated to prevent or ameliorate the development of immune mediated diseases such as AAU and SpAs.

## Immune Mechanisms and Anterior Uveitis

Immune mechanisms involved in the pathogenesis of AU have been investigated in human and animal studies. It was recently reported that in patients with HLA B27 AAU the aqueous humor concentration of CC chemokines, including CCL8, CCL13, and CCL20, were upregulated, compared with patients with granulomatous uveitis and control subjects (51). Such chemokines play a key role in attracting inflammatory cells to the eye during attacks of uveitis and may represent potential therapeutic targets.

The pro-inflammatory cytokine IL-6 has previously been shown to be produced by ocular parenchymal cells and to promote the differentiation of B cells to plasma cells (52). Recently Kumar et al. demonstrated that tear levels of IL-6 and IL-10 are elevated during acute and multiple episodes of uveitis (53) building on previous work identifying elevated IL-6 levels in human aqueous (54), vitreous and monocyte culture supernatant (55). In a study examining cytokine profiles of specific uveitic entities Abu El-Asrar et al. discovered elevated GM-CSF, IL-11, and IL-35 levels in HLA B27 AAU (56). IL-11 is classed within the IL-6 family, based on its shared use of the glycoprotein-130 receptor β-subunit (57) and in the study had a particularly strong association with disease activity. The coupled elevation of inhibitory cytokines IL-35 and IL-10 with IL-6 and IL-ll may represent an immunoregulatory response in uveitis (53, 56).

A recent study by Zhao et al. reported increased levels of IL37 mRNA and protein expression by peripheral blood mononuclear cells from patients with active AAU compared with control (58). There was no difference between HLA B27 positive and negative patients with AAU. Interestingly IL37 was shown to inhibit the production of pro-inflammatory cytokines, including IL-1, IL-6, IL-10, IL-21, IL-23, TNF, and gamma interferon in the supernatant of dendritic cells (58). Thus, IL37 may inhibit pro-inflammatory cytokines in patients with AAU and may be a new therapeutic approach to treating this disease.

It has previously been demonstrated that HLA B27 homodimers on the surface of T-cells, monocytes and natural killer cells may trigger innate immune mechanisms through binding to killer immunoglobulin-like receptors (KIR) (59). Specifically, KIR3DL2 receptor expression has been found to be increased on NK cells in the peripheral blood of patients with HLA B27 AAU, associated with higher active numbers and increased cytotoxicity (60). Using flow cytometric methods Huang et al. have now also implicated mucosa associated innate lymphoid (MAIT) cells, which in the study produced more IL-17A than controls (61), an important proinflammatory cytokine seen in HLA B27 AAU (62, 63).

In a comparison of peripheral monocytes in patients with idiopathic uveitis against those with HLA B27 AAU using ELISA methods, increased expression of S100A8/A9 was found in patients with HLA B27 AAU outside of uveitis episodes accompanied by heightened numbers and activity of monocytes and T cells (64).

## New Developments in the Treatment of HLA B27 Anterior Uveitis

A better understanding of the pathogenesis of AAU will provide new avenues for management of uveitis with the possibility of specific therapy that can alter the natural history or even cure uveitis. At present, treatment options consist of local or systemic anti-inflammatory or immunosuppressive drugs, which may be associated with treatment limiting side effects.

Current first line therapy for acute episodes of HLA B27 AAU is a cycloplegic, typically atropine 1%, and very frequent administration of potent topical corticosteroids (1–2 hourly) which are then slowly tapered over 8–10 weeks (65). The so called “steroid response” leads to elevated intraocular pressure in up to 30% of patients using topical corticosteroids, often requiring the addition of pressure lowering medication and requiring cessation of topical corticosteroid therapy in some patients. Corticosteroid therapy is also associated with increased rates of posterior subcapsular cataract (66). Although a large randomized clinical trial of 193 subjects failed to achieve non-inferiority of iontophoresis as a method to increase anterior chamber corticosteroid levels compared to topical therapy, there was a promising signal that should be tested using larger data sets (67).

In patients with vision threatening uveitis, periocular corticosteroid injections are safe and efficacious, either alone or as adjuncts to systemic therapies (68). Peri- and intra-ocular injections reduce macular oedema which is the major inflammatory mediated cause of visual loss in uveitis patients (69). Regional corticosteroid therapies have a relatively short duration of effect necessitating 3 to 6 monthly injections. The development of both bioerodible (Iluvien) and non-biodegradable (Retisert) intravitreal implants of polymers allow the controlled release of fluocinolone acetate into the vitreous for up to 3 years. Both the multicenter uveitis steroid treatment (MUST) study and a recent Cochrane review that compared Retisert implants to systemic combination immunosuppressive therapy, showed that systemic therapy resulted in superior visual outcomes and lower rates of raised intraocular pressure, cataract formation, retinal detachment and endophthalmitis (70). There was no difference in systemic side effects such as weight gain, diabetes and hypertension in the two groups and the only significant adverse outcome was increased use of antibiotics for infections in those on systemic therapy (71). Nearly two thirds of the patients in the systemic therapy arm of the MUST study required intermittent local corticosteroids.

The systemic treatment for eye diseases (SITE) cohort study retrospectively examined the efficacy of systemic therapies in 7,957 patients across five centers from 1979 to 2005 who had non-infectious ocular inflammatory disease. For patients with AU corticosteroid sparing success (tapering to 10 mg or less) was achieved over a 6-month period in 47.2% with mycophenolate mofetil, 16.6% with azathioprine, 28.5% with cyclosporine, and 46.1% with methotrexate (72–75). The role of disease modifying agents (DMARDS) have now been examined specifically in HLA B27 AAU with methotrexate and sulfasalazine both shown to reduce relapse rates and methotrexate additionally proven to reduce AU related macular oedema (76).

There is significant evidence that anti-TNF alpha therapy is of benefit in patients with HLA B27 uveitis (77–80). In a retrospective analysis of 717 patients with SpA those who were treated with either infliximab or etanercept had 6.8 AAU recurrences per hundred patient years versus 15.6 in the placebo group (81). Data from the use of adalimumab in another cohort of 1,250 patients with SpA demonstrated an overall 51% reduction in uveitis recurrence rate (82). Further analysis of these cohorts showed that of the anti-TNF therapies available, anti-TNF antibodies appear to have greater efficacy than soluble TNF receptor blockers (83). Adding weight to this preference is a collection of case reports and new data from a large Swedish rheumatology study suggesting that in AS etanercept may induce new uveitis (84, 85). Prospective studies of anti-TNF antibodies have now been performed on small cohorts of patients with frequent and/or refractory AAU. The use of infliximab led to the complete resolution of HLA B27 AAU in 6 of 7 patients following an average of 8 days (86). The new anti-TNF alpha agent golimumab induced the remission of uveitis in 12 of 15 eyes with significant improvements in visual acuity (87). Certolizumab pegol has been used in AAU cases refractory to even previous biologic therapy and achieved quiescence in 5 of 7 patients (88).

The success of the antibiotic sulfasalazine as a DMARD in HLA B27 AAU adds weight to the prospect of targeting the microbiome for disease control. Clinical studies are currently underway evaluating faecal microbial transplants in AS and it will be interesting to see whether this influences rates of uveitis in these patients (89). If the gut microbiome does play a key role in diseases induction, a variety of therapeutic strategies could be developed to alter the microbiome or the immune response to particular microorganisms implicated to prevent or ameliorate the development of immune mediated diseases such as AAU and SpA’s.

## Advantages in Studying HLA B27 Acute Anterior Uveitis

There are significant advantages in studying the immunopathogenesis of AAU as a pathophysiological model for HLA B27 related disease. AAU is well defined disorder, that is readily and reliably diagnosed clinically by slit lamp bio-microscopy. Importantly there are agreed objective grading systems to determine the severity and changes in the grade of the protein exudation and cellular infiltration in the anterior chamber that characterize AU (90). Additionally, aqueous humor can be easily sampled for analysis. The natural history of AAU has been extensively studied and has a recurrent, relapsing and remitting course. Importantly, there is a group of subjects with a similar clinical phenotype, who are HLA B27 negative and form a relevant critical disease control group. The recurrent nature of the disease also has advantages for investigators studying environmental factors, such as the gut microbiome, that may trigger relapses of uveitis. There is a large number of patients with recurrent AAU, not on systemic therapy or immunosuppressive medication, which can influence immunological and bio-molecular investigations. As 50%–70% with HLA B27 AU have an associated systemic disease, usually a SpA or inflammatory bowel disease, subjects without confounding systemic disease can be studied and compared with subjects with systemic disease (91, 92).

## Conclusion

AU is a relatively common polygenic disease, strongly associated with HLA B27 and a number of other inflammatory diseases that share a common gene pool. The function of HLA B27 and the other recently described genes predisposing to AAU is poorly understood. The role of infection and in particular the microbiome remain active areas of research, as does the importance of innate immunity in the pathogenesis of this disease. New insights into genetic, microbial, and immune mechanisms has the potential to lead to more effective preventative and suppressive forms of therapy.

## Author Contributions

All authors contributed to writing, editing, and reviewing the manuscript. All authors contributed to the article and approved the submitted version.

## Conflict of Interest

The authors declare that the research was conducted in the absence of any commercial or financial relationships that could be construed as a potential conflict of interest.

## References

[1] Baarsma GS The epidemiology and genetics of endogenous uveitis: a review. Curr Eye Res (1992) 11 Suppl:1–9. 10.3109/02713689208999505 1424734

[2] Wakefield D Chang JH Epidemiology of Uveitis. Int Ophthalmol Clin (2005) 45:1–13. 10.1097/01.iio.0000155938.83083.94 15791154

[3] González MM Solano MM Porco TC Oldenburg CE Acharya NR Lin SC Epidemiology of uveitis in a US population-based study. J Ophthalmic Inflammation Infect (2018) 8:6. 10.1186/s12348-018-0148-5 PMC5904090 29666980

[4] Kopplin LJ Mount G Suhler EB Review for Disease of the Year: Epidemiology of HLA-B27 Associated Ocular Disorders. Ocul Immunol Inflammation (2016) 24:470–5. 10.1080/09273948.2016.1175642 PMC6733982 27232197

[5] Suttorp-Schulten MS Rothova A The possible impact of uveitis in blindness: a literature survey. Br J Ophthalmol (1996) 80:844–8. 10.1136/bjo.80.9.844 PMC505625 8962842

[6] Khan MA HLA-B27 and its pathogenic role. J Clin Rheumatol (2008) 14:50–2. 10.1097/RHU.0b013e3181637a38 18431102

[7] Martin TM Rosenbaum JT An update on the genetics of HLA-B27 associated acute anterior uveitis. Ocul Immunol Inflammation (2011) 19:108–14. 10.3109/09273948.2011.559302 PMC3083239 21428748

[8] Braun J Sieper J Ankylosing spondylitis. Lancet (2007) 369:1379–90. 10.1016/s0140-6736(07)60635-7 17448825

[9] Derakhshan MH Dean L Jones GT Macfarlane GJ Siebert S Gaffney K Op0262 Factors Associated with Acute Anterior Uveitis in Patients with Axial Spondyloarthritis: Analysis of the Bsrbr-as Register Database. Ann Rheum Dis (2019) 78:212–2. 10.1136/annrheumdis-2019-eular.2859

[10] Yang P Wan W Du L Zhou Q Qi J Liang L Clinical features of HLA-B27-positive acute anterior uveitis with or without ankylosing spondylitis in a Chinese cohort. Br J Ophthalmol (2018) 102:215. 10.1136/bjophthalmol-2016-309499 28607176

[11] Dodds EM Lowder CY Meisler DM Posterior segment inflammation in HLA-B27+ acute anterior uveitis: clinical characteristics. Ocul Immunol Inflammation (1999) 7:85–92. 10.1076/ocii.7.2.85.4015 10420203

[12] Rothova A van Veenedaal WG Linssen A Glasius E Kijlstra A de Jong PT Clinical features of acute anterior uveitis. Am J Ophthalmol (1987) 103:137–45. 10.1016/s0002-9394(14)74218-7 3492916

[13] Gritz DC Schwaber EJ Wong IG Complications of Uveitis: The Northern California Epidemiology of Uveitis Study. Ocul Immunol Inflammation (2018) 26:584–94. 10.1080/09273948.2016.1247174 28112975

[14] D’Ambrosio EM La Cava M Tortorella P Gharbiya M Campanella M Iannetti L Clinical Features and Complications of the HLA-B27-associated Acute Anterior Uveitis: A Metanalysis. Semin Ophthalmol (2017) 32:689–701. 10.3109/08820538.2016.1170158 27404944

[15] Derhaag PJ Linssen A Broekema N de Waal LP Feltkamp TE A familial study of the inheritance of HLA-B27-positive acute anterior uveitis. Am J Ophthalmol (1988) 105:603–6. 10.1016/0002-9394(88)90051-7 3259836

[16] Robinson PC Claushuis TA Cortes A Martin TM Evans DM Leo P Genetic dissection of acute anterior uveitis reveals similarities and differences in associations observed with ankylosing spondylitis. Arthritis Rheumatol (2015) 67:140–51. 10.1002/art.38873 PMC4302162 25200001

[17] Huang X-F Li Z De Guzman E Robinson P Gensler L Ward MM Genomewide Association Study of Acute Anterior Uveitis Identifies New Susceptibility Loci. Invest Ophthalmol Vis Sci (2020) 61. 10.1167/iovs.61.6.3 PMC7415282 32492107

[18] Barnea E Melamed Kadosh D Haimovich Y Satumtira N Dorris ML Nguyen MT The Human Leukocyte Antigen (HLA)-B27 Peptidome in Vivo, in Spondyloarthritis-susceptible HLA-B27 Transgenic Rats and the Effect of Erap1 Deletion. Mol Cell Proteomics MCP (2017) 16:642–62. 10.1074/mcp.M116.066241 PMC5383784 28188227

[19] Faham M Carlton V Moorhead M Zheng J Klinger M Pepin F Discovery of T Cell Receptor beta Motifs Specific to HLA-B27-Positive Ankylosing Spondylitis by Deep Repertoire Sequence Analysis. Arthritis Rheumatol (2017) 69:774–84. 10.1002/art.40028 28002888

[20] Savolainen E Kettunen A Narvanen A Kautiainen H Karkkainen U Luosujarvi R Prevalence of antibodies against Chlamydia trachomatis and incidence of C. trachomatis-induced reactive arthritis in an early arthritis series in Finland in 2000. Scand J Rheumatol (2009) 38:353–6. 10.1080/03009740902769559 19296404

[21] Carter JD Gerard HC Espinoza LR Ricca LR Valeriano J Snelgrove J Chlamydiae as etiologic agents in chronic undifferentiated spondylarthritis. Arthritis Rheum (2009) 60:1311–6. 10.1002/art.24431 PMC2757404 19404948

[22] Girschick HJ Guilherme L Inman RD Latsch K Rihl M Sherer Y Bacterial triggers and autoimmune rheumatic diseases. Clin Exp Rheumatol (2008) 26:S12–7. 18570749

[23] Siala M Gdoura R Younes M Fourati H Cheour I Meddeb N Detection and frequency of Chlamydia trachomatis DNA in synovial samples from Tunisian patients with reactive arthritis and undifferentiated oligoarthritis. FEMS Immunol Med Microbiol (2009) 55:178–86. 10.1111/j.1574-695X.2008.00524.x 19159429

[24] Kuipers JG Sibilia J Bas S Gaston H Granfors K Vischer TL Reactive and undifferentiated arthritis in North Africa: use of PCR for detection of Chlamydia trachomatis. Clin Rheumatol (2009) 28:11–6. 10.1007/s10067-008-0968-z 18688674

[25] Banicioiu-Covei S Vreju AF Ciurea PL Correlations between infection patterns and eye damage in reactive arthritis. Rom J Morphol Embryol (2019) 60:601–4. 31658334

[26] Maillet J Ottaviani S Tubach F Roy C Nicaise-Rolland P Palazzo E Anti-Saccharomyces cerevisiae antibodies (ASCA) in spondyloarthritis: Prevalence and associated phenotype. Joint Bone Spine (2016) 83:665–8. 10.1016/j.jbspin.2015.10.011 26992953

[27] Wakefield D Easter J Robinson P Graham D Penny R Chlamydial antibody crossreactivity with peripheral blood mononuclear cells of patients with ankylosing spondylitis: the role of HLA B27. Clin Exp Immunol (1986) 63:49–57. 3485486 PMC1577339

[28] Scofield RH Kurien B Gross T Warren WL Harley JB HLA-B27 binding of peptide from its own sequence and similar peptides from bacteria: implications for spondyloarthropathies. Lancet (1995) 345:1542–4. 10.1016/s0140-6736(95)91089-1 7791441

[29] Schwimmbeck PL Oldstone MB Molecular mimicry between human leukocyte antigen B27 and Klebsiella. Consequences for spondyloarthropathies. Am J Med (1988) 85:51–3. 10.1016/0002-9343(88)90385-3 2462350

[30] Lahesmaa R Skurnik M Vaara M Leirisalo-Repo M Nissila M Granfors K Molecular mimickry between HLA B27 and Yersinia, Salmonella, Shigella and Klebsiella within the same region of HLA alpha 1-helix. Clin Exp Immunol (1991) 86:399–404. 10.1111/j.1365-2249.1991.tb02944.x 1747948 PMC1554211

[31] Horai R Zárate-Bladés CR Dillenburg-Pilla P Chen J Kielczewski JL Silver PB Microbiota-Dependent Activation of an Autoreactive T Cell Receptor Provokes Autoimmunity in an Immunologically Privileged Site. Immunity (2015) 43:343–53. 10.1016/j.immuni.2015.07.014 PMC4544742 26287682

[32] Schittenhelm RB Sivaneswaran S Lim Kam Sian TCC Croft NP Purcell AW Human Leukocyte Antigen (HLA) B27 Allotype-Specific Binding and Candidate Arthritogenic Peptides Revealed through Heuristic Clustering of Data-independent Acquisition Mass Spectrometry (DIA-MS) Data. Mol Cell Proteomics MCP (2016) 15:1867–76. 10.1074/mcp.M115.056358 PMC5083097 26929215

[33] Schittenhelm RB Sian TC Wilmann PG Dudek NL Purcell AW Revisiting the arthritogenic peptide theory: quantitative not qualitative changes in the peptide repertoire of HLA-B27 allotypes. Arthritis Rheumatol (2015) 67:702–13. 10.1002/art.38963 25418920

[34] Rosenbaum JT Asquith M The Microbiome and HLA B27-associated acute anterior uveitis. Nat Rev Rheumatol (2018) 14:704–13. 10.1038/s41584-018-0097-2 PMC6597169 30301938

[35] Clemente JC Manasson J Scher JU The role of the gut microbiome in systemic inflammatory disease. BMJ (2018) 360. 10.1136/bmj.j5145 PMC6889978 29311119

[36] Rosenbaum JT Lin P Asquith M Does the microbiome cause B27-related acute anterior uveitis? Ocul Immunol Inflammation (2016) 24:440–4. 10.3109/09273948.2016.1142574 PMC4988830 27002532

[37] Lin P Bach M Asquith M Lee AY Akileswaran L Stauffer P HLA-B27 and Human β2-Microglobulin Affect the Gut Microbiota of Transgenic Rats. PloS One (2014) 9. 10.1371/journal.pone.0105684 PMC4139385 25140823

[38] Colmegna I Cuchacovich R Espinoza LR HLA-B27-Associated Reactive Arthritis: Pathogenetic and Clinical Considerations. Clin Microbiol Rev (2004) 17:348–69. 10.1128/CMR.17.2.348-369.2004 PMC387405 15084505

[39] Hoekzema R Verhagen C van HM Kijlstra A Endotoxin-induced uveitis in the rat. The significance of intraocular interleukin-6. Invest Ophthalmol Vis Sci (1992) 33:532–9. 1544781

[40] Chang JH Hampartzoumian T Everett B Lloyd A McCluskey PJ Wakefield D Changes in Toll-like Receptor (TLR)-2 and TLR4 Expression and Function but Not Polymorphisms Are Associated with Acute Anterior Uveitis. Invest Ophthalmol Vis Sci (2007) 48:1711–7. 10.1167/iovs.06-0807 17389503

[41] Cusick MF Libbey JE Fujinami RS Molecular Mimicry as a Mechanism of Autoimmune Disease. Clin Rev Allergy Immunol (2012) 42:102–11. 10.1007/s12016-011-8294-7 PMC3266166 22095454

[42] Luo A Leach ST Barres R Hesson LB Grimm MC Simar D The Microbiota and Epigenetic Regulation of T Helper 17/Regulatory T Cells: In Search of a Balanced Immune System. Front Immunol (2017) 8:417. 10.3389/fimmu.2017.00417 28443096 PMC5385369

[43] Bodis G Toth V Schwarting A Role of Human Leukocyte Antigens (HLA) in Autoimmune Diseases. Rheumatol Ther (2018) 5:5–20. 10.1007/s40744-018-0100-z 29516402 PMC5935613

[44] Wakefield D Stahlberg TH Toivanen A Granfors K Tennant C Serologic evidence of Yersinia infection in patients with anterior uveitis. Arch Ophthalmol (1990) 108:219–21. 10.1001/archopht.1990.01070040071033 2302105

[45] Geczy AF Alexander K Bashir HV Edmonds J A factor(s) in Klebsiella culture filtrates specifically modifies an HLA-B27 associated cell-surface component. Nature (1980) 283:782–4. 10.1038/283782a0 7354870

[46] Scofield RH Warren WL Koelsch G Harley JB A hypothesis for the HLA-B27 immune dysregulation in spondyloarthropathy: contributions from enteric organisms, B27 structure, peptides bound by B27, and convergent evolution. Proc Natl Acad Sci USA (1993) 90:9330–4. 10.1073/pnas.90.20.9330 PMC47561 8415702

[47] Amjadi S Robertson P McCluskey P Wakefield D The role of Chlamydia in HLA-B27 Acute Anterior Uveitis and Seronegative Arthritis Amjadi S, Robertson P, McCluskey PJ, Wakefield D. Invest Ophthalmol Vis Sci (2015) 56:1847.

[48] Kuon W Kuhne M Busch DH Atagunduz P Seipel M Wu P Identification of novel human aggrecan T cell epitopes in HLA-B27 transgenic mice associated with spondyloarthropathy. J Immunol (2004) 173:4859–66. 10.4049/jimmunol.173.8.4859 15470026

[49] Tsuchiya N Williams Jr.RC Molecular mimicry–hypothesis or reality? West J Med (1992) 157:133–8. PMC1011230 1279899

[50] Wen X Hu X Miao L Ge X Deng Y Bible PW Epigenetics, microbiota, and intraocular inflammation: New paradigms of immune regulation in the eye. Prog Retin Eye Res (2018) 64:84–95. 10.1016/j.preteyeres.2018.01.001 29357307

[51] Abu El-Asrar AM Berghmans N Al-Obeidan SA Gikandi PW Opdenakker G Van Damme J The CC chemokines CCL8, CCL13 and CCL20 are local inflammatory biomarkers of HLA-B27-associated uveitis. Acta Ophthalmol (Copenh) (2019) 97:e122–8. 10.1111/aos.13835 30242977

[52] Horai R Caspi RR Cytokines in autoimmune uveitis. J Interferon Cytokine Res (2011) 31:733–44. 10.1089/jir.2011.0042 PMC3189550 21787221

[53] Kumar A Sharma SP Agarwal A Gupta V Katoch D Sehgal S Tear IL-6 and IL-10 levels in HLA-B27-Associated Uveitis and Its clinical Implications. Ocul Immunol Inflammation (2020) 28:1–7. 10.1080/09273948.2019.1704022 31940227

[54] Abu El-Asrar AM Berghmans N Al-Obeidan SA Mousa A Opdenakker G Van Damme J The Cytokine Interleukin-6 and the Chemokines CCL20 and CXCL13 Are Novel Biomarkers of Specific Endogenous Uveitic Entities. Invest Ophthalmol Vis Sci (2016) 57:4606–13. 10.1167/iovs.16-19758 27603722

[55] Perez VL Papaliodis GN Chu D Anzaar F Christen W Foster CS Elevated levels of interleukin 6 in the vitreous fluid of patients with pars planitis and posterior uveitis: the Massachusetts eye & ear experience and review of previous studies. Ocul Immunol Inflammation (2004) 12:193–201. 10.1080/092739490500282 15385196

[56] Abu El-Asrar AM Berghmans N Al-Obeidan SA Gikandi PW Opdenakker G Van Damme J Local Cytokine Expression Profiling in Patients with Specific Autoimmune Uveitic Entities. Ocul Immunol Inflammation (2019) 27:1–10. 10.1080/09273948.2019.1604974 31161935

[57] Murakami M Kamimura D Hirano T New IL-6 (gp130) family cytokine members, CLC/NNT1/BSF3 and IL-27. Growth Factors (2004) 22:75–7. 10.1080/08977190410001715181 15253382

[58] Zhao B Zhang S Chen W Zhang R Jiang R Zhang R Elevated Interleukin 37 Expression Associated With Disease Activity in HLA-B27 Associated Anterior Uveitis and Idiopathic Anterior Uveitis. Curr Mol Med (2018) 17:460–7. 10.2174/1566524018666180207152941 29424310

[59] Kuon W Holzhutter HG Appel H Grolms M Kollnberger S Traeder A Identification of HLA-B27-restricted peptides from the Chlamydia trachomatis proteome with possible relevance to HLA-B27-associated diseases. J Immunol (2001) 167:4738–46. 10.4049/jimmunol.167.8.4738 11591805

[60] Chan AT Kollnberger SD Wedderburn LR Bowness P Expansion and enhanced survival of natural killer cells expressing the killer immunoglobulin-like receptor KIR3DL2 in spondylarthritis. Arthritis Rheum (2005) 52:3586–95. 10.1002/art.21395 16255049

[61] Huang JC-C Schleisman M Choi D Mitchell C Watson L Asquith M Preliminary Report on Interleukin-22, GM-CSF, and IL-17F in the Pathogenesis of Acute Anterior Uveitis. Ocul Immunol Inflammation (2019) 27:1–8. 10.1080/09273948.2019.1686156 PMC7246145 31763950

[62] Zou W Wu Z Xiang X Sun S Zhang J The expression and significance of T helper cell subsets and regulatory T cells CD(4)(+) CD(2)(5)(+) in peripheral blood of patients with human leukocyte antigen B27-positive acute anterior uveitis. Graefes Arch Clin Exp Ophthalmol (2014) 252:665–72. 10.1007/s00417-014-2567-9 24477536

[63] El-Asrar AM Struyf S Kangave D Al-Obeidan SS Opdenakker G Geboes K Cytokine profiles in aqueous humor of patients with different clinical entities of endogenous uveitis. Clin Immunol (2011) 139:177–84. 10.1016/j.clim.2011.01.014 21334264

[64] Kasper M Walscheid K Laffer B Bauer D Busch M Wildschütz L The Phenotype of Monocytes in Anterior Uveitis Depends on the HLA-B27 Status. Front Immunol (2018) 9:1773. 10.3389/fimmu.2018.01773 30105034 PMC6077321

[65] Dunn JP Review of immunosuppressive drug therapy in uveitis. Curr Opin Ophthalmol (2004) 15:293–8. 10.1097/00055735-200408000-00003 15232467

[66] Tripathi RC Parapuram SK Tripathi BJ Zhong Y Chalam KV Corticosteroids and glaucoma risk. Drugs Aging (1999) 15:439–50. 10.2165/00002512-199915060-00004 10641955

[67] Sheppard J Garg S Lievens C Brandano L Wirostko B Korenfeld M Iontophoretic Dexamethasone Phosphate Compared to Topical Prednisolone Acetate 1% for Noninfectious Anterior Segment Uveitis. Am J Ophthalmol (2020) 211:76–86. 10.1016/j.ajo.2019.10.032 31726034

[68] Thorne JE Sugar EA Holbrook JT Burke AE Altaweel MM Vitale AT Multicenter Uveitis Steroid Treatment Trial Research Group. Periocular Triamcinolone vs. Intravitreal Triamcinolone vs. Intravitreal Dexamethasone Implant for the Treatment of Uveitic Macular Edema: The PeriOcular vs. INTravitreal corticosteroids for uveitic macular edema (POINT) Trial. Ophthalmology (2019) 126:283–95. 10.1016/j.ophtha.2018.08.021 PMC6348060 30269924

[69] Rothova A Suttorp-van Schulten MS Frits Treffers W Kijlstra A Causes and frequency of blindness in patients with intraocular inflammatory disease. Br J Ophthalmol (1996) 80:332–6. 10.1136/bjo.80.4.332 PMC505460 8703885

[70] Brady CJ Villanti AC Law HA Rahimy E Reddy R Sieving PC Corticosteroid implants for chronic non-infectious uveitis. Cochrane Database Syst Rev (2016) 2016(2). 10.1002/14651858.CD010469.pub2 PMC5038923 26866343

[71] Kempen JH Altaweel MM Holbrook JT Sugar EA Thorne JE Jabs DA Association Between Long-Lasting Intravitreous Fluocinolone Acetonide Implant vs Systemic Anti-inflammatory Therapy and Visual Acuity at 7 Years Among Patients With Intermediate, Posterior, or Panuveitis. JAMA (2017) 317:1993–2005. 10.1001/jama.2017.5103 28477440 PMC5540027

[72] Gangaputra S Newcomb CW Liesegang TL Kaçmaz RO Jabs DA Levy-Clarke GA Methotrexate for ocular inflammatory diseases. Ophthalmology (2009) 116:2188–98.e1. 10.1016/j.ophtha.2009.04.020 PMC3785935 19748676

[73] Daniel E Thorne JE Newcomb CW Pujari SS Kaçmaz RO Levy-Clarke GA Mycophenolate Mofetil for Ocular Inflammation. Am J Ophthalmol (2010) 149:423–32. 10.1016/j.ajo.2009.09.026 PMC2826576 20042178

[74] Kaçmaz RO Kempen JH Newcomb C Daniel E Gangaputra S Nussenblatt RB Cyclosporine for Ocular Inflammatory Diseases. Ophthalmology (2010) 117:576–84. 10.1016/j.ophtha.2009.08.010 PMC2830390 20031223

[75] Pasadhika S Kempen JH Newcomb C Liesegang TL Pujari SS Rosenbaum JT Azathioprine for Ocular Inflammatory Diseases. Am J Ophthalmol (2009) 148:500–9.e2. 10.1016/j.ajo.2009.05.008 19570522 PMC2753718

[76] zu Hoerste MM Walscheid K Tappeiner C Zurek-Imhoff B Heinz C Heiligenhaus A The effect of methotrexate and sulfasalazine on the course of HLA-B27-positive anterior uveitis: results from a retrospective cohort study. Graefes Arch Clin Exp Ophthalmol (2018) 256:1985–92. 10.1007/s00417-018-4082-x 30069748

[77] Dick AD Duncan L Hale G Waldmann H Isaacs J Neutralizing TNF-alpha activity modulates T-cell phenotype and function in experimental autoimmune uveoretinitis. J Autoimmun (1998) 11:255–64. 10.1006/jaut.1998.0197 9693974

[78] Dick AD Forrester JV Liversidge J Cope AP The role of tumour necrosis factor (TNF-alpha) in experimental autoimmune uveoretinitis (EAU). Prog Retin Eye Res (2004) 23:617–37. 10.1016/j.preteyeres.2004.06.005 15388077

[79] Khera TK Dick AD Nicholson LB Mechanisms of TNFα regulation in uveitis: focus on RNA-binding proteins. Prog Retin Eye Res (2010) 29:610–21. 10.1016/j.preteyeres.2010.08.003 20813201

[80] Giraudo E Primo L Audero E Gerber HP Koolwijk P Soker S Tumor necrosis factor-alpha regulates expression of vascular endothelial growth factor receptor-2 and of its co-receptor neuropilin-1 in human vascular endothelial cells. J Biol Chem (1998) 273:22128–35. 10.1074/jbc.273.34.22128 9705358

[81] Braun J Baraliakos X Listing J Sieper J Decreased incidence of anterior uveitis in patients with ankylosing spondylitis treated with the anti-tumor necrosis factor agents infliximab and etanercept. Arthritis Rheum (2005) 52:2447–51. 10.1002/art.21197 16052578

[82] Rudwaleit M Rødevand E Holck P Vanhoof J Kron M Kary S Adalimumab effectively reduces the rate of anterior uveitis flares in patients with active ankylosing spondylitis: results of a prospective open-label study. Ann Rheum Dis (2008) 68:696–701. 10.1136/ard.2008.092585 18662932 PMC2663712

[83] Guignard S Gossec L Salliot C Ruyssen-Witrand A Luc M Duclos M Efficacy of tumour necrosis factor blockers in reducing uveitis flares in patients with spondylarthropathy: a retrospective study. Ann Rheum Dis (2006) 65:1631–4. 10.1136/ard.2006.052092 PMC1798480 16901960

[84] Reddy AR Backhouse OC Does etanercept induce uveitis? Br J Ophthalmol (2003) 87:925. 10.1136/bjo.87.7.925 PMC1771735 12812909

[85] Lie E Lindström U Zverkova-Sandström T Olsen IC Forsblad-d’Elia H Askling J Tumour necrosis factor inhibitor treatment and occurrence of anterior uveitis in ankylosing spondylitis: results from the Swedish biologics register. Ann Rheum Dis (2017) 76:1515–21. 10.1136/annrheumdis-2016-210931 28254789

[86] El-Shabrawi Y Hermann J Anti-tumor necrosis factor-alpha therapy with infliximab as an alternative to corticosteroids in the treatment of human leukocyte antigen B27-associated acute anterior uveitis. Ophthalmology (2002) 109:2342–6. 10.1016/s0161-6420(02)01292-7 12466181

[87] Yazgan S Celik U Işık M Yeşil NK Baki AE Şahin H Efficacy of golimumab on recurrent uveitis in HLA-B27-positive ankylosing spondylitis. Int Ophthalmol (2017) 37:139–45. 10.1007/s10792-016-0239-y 27154720

[88] Llorenç V Mesquida M de la Maza MS Blanco R Calvo V Maíz O Certolizumab Pegol, a New Anti-TNF-α in the Armamentarium against Ocular Inflammation. Ocul Immunol Inflammation (2016) 24:167–72. 10.3109/09273948.2014.967779 25325834

[89] Hiltunen J The Role of Gut Microbiota in the Pathogenesis of Ankylosing Spondylitis (AS), and the Effect of Fecal Microbiota Transplantation on Gut Microbiota, Gut Wall Inflammation and Clinical Activity of AS. clinicaltrials.gov (2019). Available at: https://clinicaltrials.gov/ct2/show/NCT03726645 (Accessed November 5, 2020).

[90] Jabs DA Nussenblatt RB Rosenbaum JT Standardization of Uveitis Nomenclature (SUN) Working Group. Standardization of uveitis nomenclature for reporting clinical data. Results of the First International Workshop. Am J Ophthalmol (2005) 140:509–16. 10.1016/j.ajo.2005.03.057 PMC8935739 16196117

[91] Park SC Ham D-I Clinical Features and Prognosis of HLA-B27 Positive and Negative Anterior Uveitis in a Korean Population. J Korean Med Sci (2009) 24:722–8. 10.3346/jkms.2009.24.4.722 PMC2719208 19654959

[92] Barisani-Asenbauer T Maca SM Mejdoubi L Emminger W Machold K Auer H Uveitis- a rare disease often associated with systemic diseases and infections- a systematic review of 2619 patients. Orphanet J Rare Dis (2012) 7:57. 10.1186/1750-1172-7-57 22932001 PMC3503654

